# 3D Underwater Uncooperative Target Tracking for a Time-Varying Non-Gaussian Environment by Distributed Passive Underwater Buoys

**DOI:** 10.3390/e23070902

**Published:** 2021-07-15

**Authors:** Xianghao Hou, Jianbo Zhou, Yixin Yang, Long Yang, Gang Qiao

**Affiliations:** 1 School of Marine Science and Technology, Northwestern Polytechnical University, Xi’an 710072, China; houxianghao1990@163.com (X.H.); yxyang@nwpu.edu.cn (Y.Y.); lyang@nwpu.edu.cn (L.Y.); 2 College of Underwater Acoustic Engineering, Harbin Engineering University, Harbin 150001, China; qiaogang@hrbeu.edu.cn; 3 Shaanxi Key Laboratory of Underwater Information Technology, Xi’an 710072, China

**Keywords:** underwater target tracking, adaptive tracking, particle filter, passive tracking

## Abstract

Accurate 3D passive tracking of an underwater uncooperative target is of great significance to make use of the sea resources as well as to ensure the safety of our maritime areas. In this paper, a 3D passive underwater uncooperative target tracking problem for a time-varying non-Gaussian environment is studied. Aiming to overcome the low observability drawback inherent in the passive target tracking problem, a distributed passive underwater buoys observing system is considered and the optimal topology of the distributed measurement system is designed based on the nonlinear system observability analysis theory and the Cramer–Rao lower bound (CRLB) analysis method. Then, considering the unknown underwater environment will lead to time-varying non-Gaussian disturbances for both the target’s dynamics and the measurements, the robust optimal nonlinear estimator, namely the adaptive particle filter (APF), is proposed. Based on the Bayesian posterior probability and Monte Carlo techniques, the proposed algorithm utilizes the real-time optimal estimation technique to calculate the complex noise online and tackle the underwater uncooperative target tracking problem. Finally, the proposed algorithm is tested by simulated data and comprehensive comparisons along with detailed discussions that are made to demonstrate the effectiveness of the proposed APF.

## 1. Introduction

The marine resources have significant influences on human’s living and social developments. Furthermore, the ocean plays an important role for national security and the underwater target tracking is one of the most significant research areas of marine science and underwater acoustic engineering nowadays. Therefore, to exploit the ocean resources and defend the national territory, the technology of accurately and reliably tracking an uncooperative underwater target is of vital importance.

There are two categories of underwater target tracking techniques, namely the active underwater target tracking and the passive underwater target tracking. The former always utilizes high-power active sonar equipment to transmit active detection signals to track the target in real time [1,2]. This kind of tracking technique is usually participated in the relatively close-range tracking scenarios with fixed ship-borne or shore-based sonar platforms for the limitation of power supplies. Hence, the active underwater tracking system lacks mobility and is easy to be discovered by the uncooperative target or third-party surveillance systems so that the target can make maneuvers to avoid the detections. Considering the increasingly complex modern underwater attacking and defensive environment, the passive tracking techniques seized the attentions from many researchers [3,4,5]. Passive tracking techniques only utilize the radiation noise from the target to make parameter estimations without sending any detecting signals during the tracking period. Therefore, the passive tracking sonar and the combined tracking techniques are of great invisibility and have many outstanding advantages such as a long detecting range, little power consumption, and flexible deployment constrains when compared to the active ones. Consequently, considering the large sea area to be monitored and the increasingly intelligent underwater target in the research area of underwater target tracking, the high safety coefficient and low energy consumption advanced passive tracking techniques are of great importance to be researched.

For the aspect of the passive sonar tracking system, two kinds of measurements are commonly utilized to track an uncooperative underwater target. The first one is the angle-based measurements. By collecting the passive signals from an underwater target, various kinds of DOA algorithms can be utilized to output both the azimuth angle and the elevation angle of the target [6,7]. Then, considering the angle information as the measurements, the locations and velocities of an underwater target can be estimated by various tracking algorithms. Besides the angle measurements, the frequency measurements (also known as the Doppler frequency measurements) can also provide extra information about the underwater target and make the tracking process more accurate [8]. However, for a slowly-moving underwater target, the frequency measurements are usually hard to utilize. Thus, to track a slowly-moving underwater target (such as the submarines), the angle information is the only measurement that can be utilized in the tracking procedure.

In order to build a robust and accurate underwater passive tracking system, the observability along with the accuracy analysis should be firstly studied comprehensively. Since the passive tracking system only utilizes the radiations from the interested underwater target to accomplish the tracking process and the frequency-based measurements are not available when the target moves at a slow speed far from the tracking system, only the angle information can be obtained and utilized. Therefore, the passive tracking process sometimes is called the bearing-only tracking in a 2D scenario and angle-only tracking in a 3D scenario. The observability analyses are comprehensively studied by several researchers [9,10,11], and the conclusion is made that the angle-only measurements by a single static observer is not sufficient to guarantee the system to be fully observable. In order to make a tracking system observable to satisfy the robustness requirements of the passive tracking system, the single observer must maneuver with much more agility than the target or more observers are needed. However, in the scenario of passive underwater uncooperative target tracking, it is impossible to know the target’s motion details before the tracking procedure is accomplished. Therefore, the only way of deploying more observers is realizable. In addition, since the commonly used sonar arrays must be towed or mounted by a submarine or a ship, which is hard to make concealed passive tracking networks for realistic reasons during the underwater uncooperative target tracking process, the passive distributed underwater buoys system is developed to tackle this problem. By deploying several passive underwater buoys in an interested sea area to different depth, an uncooperative target can be tracked with full 3D observability if combined angle-only measurements are utilized. Therefore, developing passive tracking techniques which depend on the distributed underwater buoys has attracted attention from various researchers and rich research outcomes have been obtained [12,13,14]. Nevertheless, there are also several crucial issues to be further studied.

Firstly, the observability of a tracking system is not “constant”. For the nonlinear system of underwater target tracking, even a full observable tracking system has different levels of observability for different configurations of the distributed underwater buoys that participate in the current tracking process. According to ref. [15], the observable degree is an important index to measure the extent to which a nonlinear tracking system is observable. For a certain passive distributed underwater buoys system, different topology of the buoys providing measurements during every tracking step can have a different observable degree. As a result, choosing certain sets of buoys to form the optimal topology of the distributed underwater passive buoys system, depending on real time tracking results, can make the tracking system more robust so that the topology designing criterion needs further investigations.

In addition, for a distributed system containing several underwater buoys, it is understandable that more measurements being utilized at the same tracking time can provide more accurate tracking results. However, since the underwater acoustic communicating resources are very limited, more underwater buoys will cost more underwater acoustic communicating resources which, as a result, will make the information fusion procedure much longer and decrease the tracking accuracy at its expense. Hence, the goal is to utilize an appropriate number of buoys to satisfy the accuracy requirements and save underwater acoustic communicating resources in the meantime. Therefore, the method of balancing the tracking accuracy and the system observability is crucial for the passive distributed underwater buoys system and has great researching significance. Although some researchers have developed the sensor scheduling and control principle for the distributed radar systems [16,17,18], the sensor selection and topology design of an underwater distributed tracking system are still open questions that attracted a number of researchers [13,14].

Besides the considerations of the measurements, the target tracking algorithms also play an indispensable role in the tracking procedure. Since the underwater uncooperative target always stays in a certain moving mode for a long time and seldom makes maneuvering, the kinematics of the target can be modeled as the constant velocity (CV) model like many researchers supposed in ref. [13]. Hence, the passive tracking problem will be focused on the nonlinear tracking algorithms design under the determined target model and measurement model. According to the scientific and engineering requirements of building the advanced passive underwater target tracking system, many researchers proposed their innovative tracking schemes [19,20,21,22]. Among all the existing underwater target tracking algorithms, the nonlinear Bayesian framework is the most common and effective approach. For the nonlinear underwater target tracking scenario, ref. [19] designed the extended Kalman filter (EKF) scheme-based tracking algorithm to make suboptimal passive estimations of the underwater target. Unlike the EKF using 1st order Taylor series expansion to linearize the nonlinear measurement model, ref. [20] designed the unscented Kalman filter (UKF) scheme-based underwater target tracking algorithms to have the theoretical accuracy enhanced to 2nd order Taylor series expansion. Moreover, in order to obtain more accurate estimations, some other more complex algorithms are proposed. The cubature Kalman filter (CKF)-based target tracking algorithm is designed by ref. [21] along with the sigma-point Kalman filter with interpolation which is proposed by ref. [22]. Although the rich researching achievements in the underwater target tracking filed, they all assume the noise to be Gaussian white noise and most of them consider the noise are time-invariant. However, for the underwater target tracking scenario, the unknown underwater environment will probably influence the kinematics of the target and the measurements made by every underwater buoy so that the noise can be time-varying and non-Gaussian. Considering this assumption, only a limited number of particle filter (PF)-based researches that utilize the Bayesian posterior estimation method to overcome the Gaussian white noise limitation are designed [23,24], and very few researchers pay attention to the time-varying characteristic of the underwater noise.

Considering all the above-mentioned challenges and in order to track the underwater uncooperative target in a 3D real-time scenario under the time-varying non-Gaussian environment by distributed passive submerged buoys, this paper firstly proposed a real-time optimal topology-forming method of the distributed measurement system that balances the tracking robustness and accuracy. Then, a robust algorithm that combined the advantages of both the Bayesian posterior estimation techniques and the online noise estimation techniques is designed. The contributions can be summarized as follows:

1. By utilizing the CRLB and FIM analysis method along with the nonlinear observability analysis techniques, a real-time optimal topology design method of the distributed passive underwater buoys is proposed to balance the estimation robustness and accuracy dynamically.
2. An online noise estimator is proposed based on the Sage-Husa estimating technique to estimate the 1st order and 2nd order momentum of the time-varying noise dynamically.
3. An intelligent tracking algorithm for a time-varying non-Gaussian environment is proposed, namely the adaptive particle filter (APF). The proposed algorithm guarantees the convergence of underwater target tracking accuracy.

The rest of this paper is organized as follows. In Section 2, the problem is formulated by introducing the kinematics of the underwater target and the measurement model of the distributed passive buoys. In Section 3, the optimal topology design method of the distributed underwater buoys based on the CRLB and FIM analysis method along with the observability analysis technique are described in detail. The innovative nonlinear tracking algorithm for the time-varying non-Gaussian environment, namely the APF, is proposed in Section 4. Section 5 gives the comprehensive simulating results and discussions to verify the effectiveness of the designed algorithm. Finally, in Section 6, the conclusions are drawn.

## 2. Underwater Target Kinematics Model and Distributed Measurement Model

In this section, the kinematics model of the underwater target and the measurement model of the distributed buoys system are proposed. Assuming the underwater uncooperative target performs a constant velocity (CV) mode, the underwater uncooperative target usually remains at the same speed to save energy. In addition, the measurements of the distributed underwater buoys are the passive angle-only information which can be divided into the azimuth angle information and the elevation angle information. The following section gives the details of the above-mentioned models, respectively.

### 2.1. Kinematic Model of the Underwater Uncooperative Target

The main motive of the 3D tracking problem considered in this work is to estimate the position and velocity of an underwater uncooperative target with CV motion mode using noise-corrupted angle-only measurements from the passive distributed underwater buoys system. Consider $\left[\begin{array}{ccc} x & y & z \end{array}\right]$ representing the current 3D location of the underwater target and $\left[\begin{array}{ccc} v_{x} & v_{y} & v_{z} \end{array}\right]$ representing the 3D velocities, respectively. According to ref. [13], the kinematics model of the underwater uncooperative target can be represented as:

(1) $$X_{k}=\Phi_{k/k-1}X_{k-1}+W_{k}$$

where $X_{k}={\left[\begin{array}{cccccc} x_{k} & y_{k} & z_{k} & v_{xk} & v_{yk} & v_{zk} \end{array}\right]}^{T}$ is the system state at tracking time k, $W_{k}$ is the time-varying non-Gaussian process noise caused by the unknown underwater environment with 1-st order and 2-nd order moments q and Q, respectively. $\Phi_{k/k-1}$ is the state transition matrix and can be represented as the following matrix if the target is in the CV operation mode:

(2) $$\Phi_{k/k-1}=\left[\begin{array}{cccccc} 1 & 0 & 0 & T & 0 & 0\\ 0 & 1 & 0 & 0 & T & 0\\ 0 & 0 & 1 & 0 & 0 & T\\ 0 & 0 & 0 & 1 & 0 & 0\\ 0 & 0 & 0 & 0 & 1 & 0\\ 0 & 0 & 0 & 0 & 0 & 1 \end{array}\right]$$

It should be noted that, unlike many researches supposing $W_{k}$ as the zero mean Gaussian white noise [13,19,20,21,22], here, $W_{k}$ is modeled as the time-varying non-Gaussian stochastic process since the unknown underwater environment always perform uncertain disturbances to the underwater uncooperative target.

### 2.2. Measurement Model of the Distributed Passive Underwater Buoys

The measurement model of the i-th buoy at tracking time k can be represented as:

(3) $$z_{i,k}=\left[\begin{array}{c} \theta_{i,k}\\ \varphi_{i,k} \end{array}\right]+V_{i,k}$$

where $\theta_{i,k}$ and $\varphi_{i,k}$ represent the measured azimuth angle and elevation angle by i-th buoy at tracking time k, respectively. Similar to the consideration of $W_{k}$ in the process model, $V_{i,k}$ is also modeled as the time-varying non-Gaussian measurement noise caused by the unknown underwater environment with 1-st order and 2-nd order moments $r_{k}$ and $R_{k}$, respectively. In this paper, we assume that all the underwater buoys have the same stochastic process of the measurement noise.

**Figure 1:**
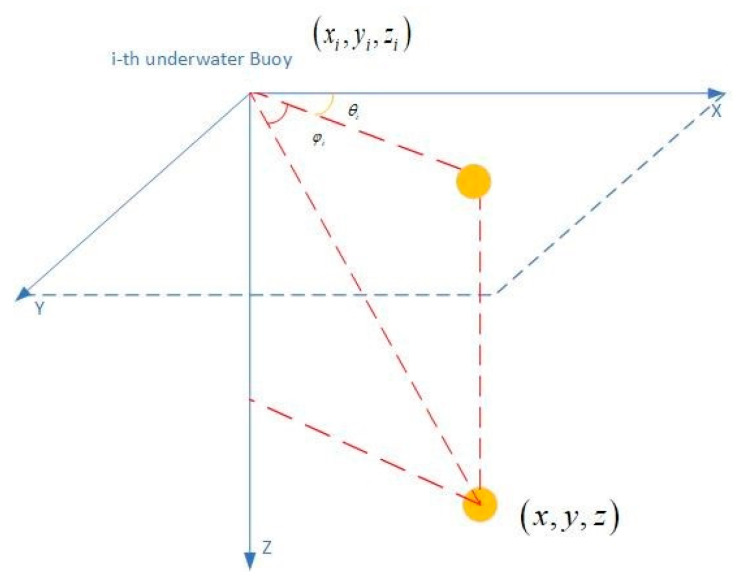
Three-dimensional measurement by *i*-th underwater buoy.

Assuming the tracking procedure is performing in the free 3D space (as shown in Figure 1), the measurement model by Equation (3) be represented in the 3D Cartesian coordinates as:

(4) $$z_{i,k}=h\left(X_{k}\right)+V_{i,k}=\left[\begin{array}{c} \arctan\frac{y_{k}-y_{i,k}}{x_{k}-x_{i,k}}\\ \arctan\frac{\sqrt{{\left(y_{k}-y_{i,k}\right)}^{2}+{\left(x_{k}-x_{i,k}\right)}^{2}}}{z_{k}-z_{i,k}} \end{array}\right]+V_{i,k}$$

where $\left(\begin{array}{ccc} x_{i,k} & y_{i,k} & z_{i,k} \end{array}\right)$ is the location of the *i*-th buoy at tracking time *k*.

## 3. Topology Design for Distributed Passive Underwater Buoys

In the section, we assume that the distributed passive tracking system contains three underwater buoys. According to the system observability analyzed by ref. [19], at least two sets of measurements need to be utilized to perform valid 3D tracking of the underwater target. In addition, limited by the constrained underwater acoustic communicating resources, performing accurate tracking by the fewest number of buoys is necessary. Therefore, in this section, an optimal criterion of topology design for the distributed passive underwater buoys system is proposed based on the Cramer–Rao lower bound (CRLB) and Fisher information matrix (FIM) analysis method along with the nonlinear observability analysis techniques. By an objective function concerning both the observability analysis results and the theoretical tracking accuracy, this section designed the optimal topology forming method of the distributed measurement system by selecting the proper buoys from the whole measurement system based on the real-time estimated the target’s states and measurements.

### 3.1. Accuracy Analysis Based on CRLB and FIM

The proposal of tracking is to estimate the target’s state accurately at every tracking step. In order to track the target as accurate as possible and utilize the underwater acoustic communication resources effectively, the accuracy evaluation method is formed by the CRLB and FIM with the measurements from two individual buoys. According to the accuracy analysis theory [19], this information can be directly obtained from the time-updated state $X_{k/k-1}$ and the location of the buoys.

From the measurement model (Equation (4)) and the system state ($X_{k}={\left[\begin{array}{cccccc} x_{k} & y_{k} & z_{k} & v_{xk} & v_{yk} & v_{zk} \end{array}\right]}^{T}$), considering there will be two individual buoys to be utilized at every tracking step, the measurements model can be expressed as:

(5) $$\begin{array}{lll} z_{ij,k} & = & \left[\begin{array}{c} \theta_{i,k}\\ \varphi_{i,k}\\ \theta_{j,k}\\ \varphi_{j,k} \end{array}\right]+V_{ij,k}\\ & = & h_{ij}(X_{k})+V_{ij,k}=\left[\begin{array}{c} \arctan\frac{y_{k}-y_{i,k}}{x_{k}-x_{i,k}}\\ \arctan\frac{\sqrt{{\left(y_{k}-y_{i,k}\right)}^{2}+{\left(x_{k}-x_{i,k}\right)}^{2}}}{z_{k}-z_{i,k}}\\ \arctan\frac{y_{k}-y_{j,k}}{x_{k}-x_{j,k}}\\ \arctan\frac{\sqrt{{\left(y_{k}-y_{j,k}\right)}^{2}+{\left(x_{k}-x_{j,k}\right)}^{2}}}{z_{k}-z_{j,k}} \end{array}\right]+V_{ij,k} \end{array}$$

where

(6) $$V_{ij,k}={\left[\begin{array}{cc} V_{i,k} & V_{j,k} \end{array}\right]}^{T}$$

is the united time-varying non-Gaussian measurement noise from the i-th and j-th buoy, and the definitions of the other parameters are the same as mentioned in Section 2.2.

From Equations (4)–(6) and the time-updated state $X_{k/k-1}$, the likelihood function of $X_{k/k-1}$ can be represented as:

(7) $$P\left(Z_{k}|X_{k/k-1}\right)=\prod_{i=1}^{2}P\left(Z_{i,k}|X_{k/k-1}\right)=\prod_{i=1}^{2}\frac{1}{2\pi }\frac{1}{\sqrt{R_{i,k}}}\exp\left\{-\frac{1}{2}{\left(Z_{i,k}-h(X_{k/k-1})\right)}^{T}R_{i,k}^{-1}\left(Z_{i,k}-h(X_{k/k-1})\right)\right\}$$

The maximum likelihood estimation-based underwater target tracking problem can be represented as maximizing the likelihood function $P\left(Z_{k}|X_{k/k-1}\right)$, that is:

(8) $${\hat{X}}_{k}=\underset{X_{k/k-1}}{\arg\max}P\left(Z_{k}|X_{k/k-1}\right)$$

Considering $L\left(X_{k/k-1}\right)=\mathrm{In}P\left(Z_{k}|X_{k/k-1}\right)$, the optimal estimation problem can be reformulated as:

(9) $${\hat{X}}_{k}=\underset{X_{k/k-1}}{\arg\max}L\left(X_{k/k-1}\right)$$

Hence, the FIM of the system formed by Equations (1) and (5) can be represented as:

(10) $$I\left(X_{k/k-1}\right)=\frac{\partial L\left(X_{k/k-1}\right)}{\partial X_{k/k-1}}$$

which leads to

(11) $$I\left(X_{k/k-1}\right)=\sum_{i=1}^{2}\frac{1}{R_{i,k}}\left[\begin{array}{cccccc} d_{ix}^{2} & d_{iy}^{}d_{ix}^{} & d_{iz}^{}d_{ix}^{} & d_{iv_{x}}^{}d_{ix}^{} & d_{iv_{y}}^{}d_{ix}^{} & d_{iv_{z}}^{}d_{ix}^{}\\ d_{iy}^{}d_{ix}^{} & d_{iy}^{2} & d_{iz}^{}d_{iy}^{} & d_{iv_{x}}^{}d_{iy}^{} & d_{iv_{y}}^{}d_{iy}^{} & d_{iv_{z}}^{}d_{iy}^{}\\ d_{iz}^{}d_{ix}^{} & d_{iy}^{}d_{iz}^{} & d_{iz}^{2} & d_{iv_{x}}^{}d_{iz}^{} & d_{iv_{y}}^{}d_{iz}^{} & d_{iv_{z}}^{}d_{iz}^{}\\ d_{iv_{x}}^{}d_{ix}^{} & d_{iv_{x}}^{}d_{iy}^{} & d_{iv_{x}}^{}d_{iz}^{} & d_{iv_{x}}^{2} & d_{iv_{y}}^{}d_{iv_{x}}^{} & d_{iv_{z}}^{}d_{iv_{x}}^{}\\ d_{iv_{y}}^{}d_{ix}^{} & d_{iv_{y}}^{}d_{iy}^{} & d_{iv_{y}}^{}d_{iz}^{} & d_{iv_{y}}^{}d_{iv_{x}}^{} & d_{iv_{y}}^{2} & d_{iv_{y}}^{}d_{iv_{z}}^{}\\ d_{iv_{z}}^{}d_{ix}^{} & d_{iv_{z}}^{}d_{iy}^{} & d_{iv_{z}}^{}d_{iz}^{} & d_{iv_{z}}^{}d_{iv_{x}}^{} & d_{iv_{z}}^{}d_{iv_{y}}^{} & d_{iv_{z}}^{2} \end{array}\right]$$

where $d_{i\zeta }=\partial h\left(X_{k/k-1}\right)/\partial \zeta$, $\zeta =X_{k/k-1}={\left[\begin{array}{cccccc} x_{k/k-1} & y_{k/k-1} & z_{k/k-1} & v_{xk/k-1} & v_{yk/k-1} & v_{zk/k-1} \end{array}\right]}^{T}$.

According to ref. [13], the minimum reachable lower bound of the estimated state’s variance is given by the CRLB, which can be represented as:

(12) $$R=E\left[\left(X_{k}-{\hat{X}}_{k}\right){\left(X_{k}-{\hat{X}}_{k}\right)}^{T}\right]\geq CRLB$$

Additionally, the CRLB can be represented by the inverse of the FIM, namely:

(13) $$CRLB≜FIM^{-1}={\left|I\left(X_{k/k-1}\right)\right|}^{-1}$$

Consequently, by minimizing the value computed by the above Equation (13), two out of three buoys of the distributed measuring system can be selected and the optimal topology based on estimating accuracy can be determined, namely:

(14) $$T_{acc}=\underset{s.t.\;i,j=\left(1,2\right)\cup i,j\left(1,3\right)\cup i,j=\left(2,3\right)}{\mathrm{argmin}}{\left|I\left(X_{k/k-1}\right)\right|}^{-1}$$

### 3.2. Observability Analysis of the Nonlinear Tracking System

Observability analysis of a nonlinear tracking system is the foundation of making reliable tracking results. From Equations (1) and (4), it can be found that the system to be analyzed is highly nonlinear. According to ref. [25], the Gramian matrix can be represented as:

(15) $$OG=\sum_{k=1}^{m}\Phi \left(t_{k},t_{0}\right)H_{k}^{T}R_{k}^{-1}H_{k}\Phi^{T}\left(t_{k},t_{0}\right)$$

where

(16) $$\Phi \left(t_{k},t_{0}\right)=\Phi_{k/k-1}\cdots \Phi_{2/1}\Phi_{1/0}$$

and *m* is the discrete tracking step during the overall tracking procedure, $H_{k}$ is the Jacobian matrix of the measurement function $h\left(X_{k}\right)$ represented by Equation (4) at tracking time k, and $R_{k}$ is the covariance matrix of the time-varying non-Gaussian measurement noise at tracking time k.

According to the linear observability theory, the states can be estimated from the measurements at any biased initial guesses when a linear system is fully observable. Hence, in order to make the tracking procedure reliable and robust, it is essential to guarantee that the tracking system is fully observable especially when the target to be tracked is uncooperative, which means that the tracking process must have the ability of convergence at any random initial guesses. However, unlike the linear system, the theory of determining whether an arbitrarily nonlinear system is fully observable is still an open question. From Equation (15), it can be found that the OG matrix is time-varying which leads to the observability of a nonlinear system having local characteristics.

According to the observability analysis criterion proposed in ref. [25], a nonlinear system is “nearly” locally weak observable when the OG matrix computed by Equation (15) at tracking time k has a full rank. Here, “nearly” locally weak observable means that the states can converge within a certain scope of initial bias but cannot guarantee that the state can be robustly estimated by any initial guesses since the OG matrix at tracking time k is computed by the Jacobian matrix of the measurement function and some deeper nonlinear features are lost [26]. For the scenario of underwater uncooperative target tracking, the prior information of the target is very limited so that the initial guesses of the states usually have randomly initial bias. Consequently, more criterions are needed besides the rank criterion for the observability analysis of the nonlinear tracking system.

Since the conditional number can reflect whether a matrix is “healthy”, the index of observable degree is introduced to reveal whether an OG matrix is better under a certain topology of the distributed buoys. The observable degree is represented as follows:

(17) $$ob=\frac{\max(\Gamma_{k})}{\min(\Gamma_{k})}$$

where $\Gamma_{k}=\left[\begin{array}{ccc} \begin{array}{cc} \lambda_{1} & \lambda_{2} \end{array} & \cdots & \lambda_{n} \end{array}\right]$ is the vector of eigenvalues of the OG matrix at tracking time *k*.

From the matrix analysis theory, the OG matrix is more robust to the disturbances when the value of ob is small. If the value of ob is very large, the maximum eigenvalue is much larger than the minimum one, which will make the OG matrix become ill-conditioned. Therefore, the relative smaller value of ob by different sets of buoys participated in the current tracking procedure indicates a better observable degree of the tracking system.

According to the above analysis, if the tracking system is locally weak observable at tracking time k, the optimal topology design criterion based on robust tracking aspect can be represented as:

(18) $$T_{ob}=\underset{s.t.\;i,j=\left(1,2\right)\cup i,j\left(1,3\right)\cup i,j=\left(2,3\right)}{\mathrm{argmin}}\left|ob\right|$$

### 3.3. Objective Function for Optimal Topology Design of the Distributed Underwater Buoys System

In order to track the underwater uncooperative target as accurate as possible and consume the least acoustic communicating resources, an unity objective function considering both the tracking accuracy and the communication usage is designed as the following:

(19) $$T_{all}=\alpha T_{acc}+(1-\alpha )T_{ob}$$

where $T_{acc}$ and $T_{ob}$ are calculated as Equations (14) and (18) under the same sets of buoys. α is the factor to adjust the relative weight of the accuracy and observability can be tuned according to the real passive tracking scenario. If more tracking accuracy is needed, the value of α can be enlarged and vice versa.

Hence, by defining the adjust parameter λ, the optimal topology design criterion of the passive distributed underwater buoys can be expressed as:

(20) $$\left(i,j\right)=\underset{s.t.\;i,j=\left(1,2\right)\cup i,j\left(1,3\right)\cup i,j=\left(2,3\right)}{\arg\min}T_{all}$$

## 4. Adaptive Tracking Algorithm for the Time-Varying Non-Gaussian Environment

From Equations (1) and (4), it can be found that the 3D passive tracking process is a high nonlinear problem with uncertain noise. Unlike many researches which regard the noise as the Gaussian white noise, we model the process noise and the measurement noise as the time-varying non-Gaussian noise since this assumption is closer to the reality. Hence, many of the existing nonlinear estimating algorithms cannot be directly adopted. To make the time-varying noise estimated online, several adaptive techniques have been developed [27,28]. However, in order to make the adaptive techniques less computational complex and easy to implement in the real underwater target tracking scenario, we introduce the modified Sage-Husa online noise-estimating technique [29] to deal with the time-varying characteristic of the noise. Then, for the nonlinear tracking problem, based on the online estimated noise, we proposed an adaptive PF (APF)-based optimal tracking algorithm to make acceptable tracking results for the time-varying non-Gaussian environment.

### 4.1. Modified Sage-Husa Online Noise Estimator

The Sage-Husa online noise estimator is firstly introduced by ref. [29] for linear system noise estimation. For the nonlinear tracking system depicted by Equations (1) and (4), the 1st and 2nd momentum of the time-varying noise at tracking time k can be online estimated by the nonlinear Sage-Husa estimator, respectively, as the following:

(21) $${\hat{q}}_{k}=\frac{1}{k}\sum_{j=1}^{k}({\hat{X}}_{j|j}-\Phi_{j/j-1}{\hat{X}}_{j|j-1})$$

(22) $${\hat{Q}}_{k}=\frac{1}{k}\sum_{j=1}^{k}[\Delta X_{j}-q]{\left[\Delta X_{j}-q\right]}^{T}$$

(23) $${\hat{r}}_{k}=\frac{1}{k}\sum_{j=1}^{k}(Z_{j}-h({\hat{X}}_{j|j}))$$

(24) $${\hat{R}}_{k}=\frac{1}{k}\sum_{j=1}^{k}[\Delta Z_{j}-r]{\left[\Delta Z_{j}-r\right]}^{T}$$

where q and r are the 1-st order momentum of the process noise and measurement noise, respectively, $\Phi_{j/j-1}$ is the transfer matrix from tracking time j−1 to j, $\Delta X_{j}$ and $\Delta Z_{j}$ are represented as:

(25) $$\Delta X_{j}={\hat{X}}_{j|j}-\Phi_{j/j-1}{\hat{X}}_{j|j-1}$$

(26) $$\Delta Z_{j}=Z_{j}-h\left({\hat{X}}_{j|j-1}\right)$$

From Equation (21) to Equation (26), it can be found that the classic nonlinear Sage-Husa online noise estimator must utilize all the smooth values of the state within a certain tracking period, which leads to the total process hard to compute. According to ref. [30], the recursive suboptimal representation of the nonlinear Sage-Husa online noise estimator can be represented as:

(27) $${\hat{q}}_{k}=\left(1-\frac{1}{k}\right){\hat{q}}_{k-1}+\frac{1}{k}\Delta X_{k}$$

(28) $${\hat{r}}_{k}=\left(1-\frac{1}{k}\right){\hat{r}}_{k-1}+\frac{1}{k}\Delta Z_{k}$$

(29) $${\hat{Q}}_{k}=\left(1-\frac{1}{k}\right){\hat{Q}}_{k-1}+\frac{1}{k}\left(K_{k}\varepsilon_{k}\varepsilon_{k}^{T}K_{k}^{T}+P_{k|k}-\Phi_{k-1}P_{k-1|k-1}\Phi_{k-1}^{T}\right)$$

(30) $${\hat{R}}_{k}={\hat{R}}_{k-1}+\frac{1}{k}\left(\varepsilon_{k}\varepsilon^{T}{}_{k}-P_{zz}{}_{,k|k-1}\right)$$

where $K_{k}$ is the filter gain by a designed tracking algorithm, $P_{k|k}$ is the covariance of the estimated state at time k, and $\varepsilon_{k}$ is the innovation represented as the following:

(31) $$\varepsilon_{k}=Z_{k}-h\left({\hat{X}}_{k|k-1}\right)-r_{k}$$

and the representation of $P_{zz}{}_{,k|k-1}$ is depended on different nonlinear tracking algorithms.

For time-varying noise, the latest measurement should be given more attention than the historical data. Therefore, the fading factor $d_{k}$ at tracking time k is introduced to the above Equations (27)–(30) as:

(32) $${\hat{q}}_{k}=\left(1-d_{k}\right){\hat{q}}_{k-1}+d_{k}\Delta X_{k}$$

(33) $${\hat{r}}_{k}=\left(1-d_{k}\right){\hat{r}}_{k-1}+d_{k}\Delta Z_{k}$$

(34) $${\hat{Q}}_{k}=\left(1-d_{k}\right){\hat{Q}}_{k-1}+d_{k}\left(K_{k}\varepsilon_{k}\varepsilon_{k}^{T}K_{k}^{T}+P_{k|k}-\Phi_{k-1}P_{k-1|k-1}\Phi_{k-1}^{T}\right)$$

(35) $${\hat{R}}_{k}=\left(1-d_{k}\right){\hat{R}}_{k-1}+d_{k}\left(\varepsilon_{k}\varepsilon^{T}{}_{k}-P_{zz}{}_{,k|k-1}\right)$$

with

(36) $$d_{k}=\frac{1-b}{1-b^{k}}$$

It can be found in Equation (36) that, when the index b is close to 1, the noise calculated by Equation (32) to Equation (35) will focus on the measurements from the total tracking period. On the contrary, if b is close to 0, the estimated noise will be more focused on the current time innovation. Consequently, by tuning the value of index b, the fading factor $d_{k}$ will be changed dynamically to affect the online noise estimation performance. Combine Equations (32)–(36), we obtain the modified Sage-Husa online noise estimator to deal with the time-varying characteristic of the process and measurement noise during the tracking procedure.

### 4.2. Adaptive Particle Filter (APF) for Underwater Target Tracking

Since the passive underwater target tracking system is highly nonlinear and the disturbances are time-varying and non-Gaussian, the classic nonlinear estimators cannot be utilized. Here, we introduce the APF to make reliable estimations under the time-varying non-Gaussian environment.

The designed APF united the modified Sage-Husa online noise-estimating technique and the PF tracking technique to track an uncooperative underwater target by a certain topology of the distributed buoys system. Depending on the nonlinear tracking system represented by Equations (1) and (5), the APF can be divided into the following steps:

1. Initialization

At tracking time k=0, selecting n particles ($x_{i},i=1\cdots n$) based on the initial guess of the state $X_{0}$ and covariance $P_{0}$. Among all the selected particles, the initial weight is set as $\frac{1}{n}$.

2. Time update

According to Equations (1) and (32), the time updated by every state particle under the time-varying non-Gaussian environment can be represented as:

(37) $$X_{k/k-1}^{i}=\Phi_{k/k-1}{\hat{X}}_{{}_{k-1}}^{i}+{\hat{q}}_{k-1}$$

where ${\hat{q}}_{k-1}$ is the 1-st order momentum of the time-varying non-Gaussian process noise $W_{k-1}$.

3. Measurement noise online estimation

According to Equations (33) and (35), the measurement noise at time k can be estimated by the modified Sage-Husa online noise estimator at every sampling time-updated state as follows:

(38) $${\hat{r}}_{k}=\left(1-d_{k}\right){\hat{r}}_{k-1}+\frac{d_{k}}{n}\left(\sum_{i=1}^{n}\left(Z_{k}-h\left(X_{k/k-1}^{i}\right)\right)\right)$$

(39) $${\hat{R}}_{k}=\left(1-d_{k}\right){\hat{R}}_{k-1}+\frac{d_{k}}{n}\sum_{i=1}^{n}\left(\varepsilon_{k}^{i}\varepsilon_{k}^{i,T}-P_{zz,k|k-1}^{i}\right)$$

where

(40) $$\varepsilon_{k}^{i}=Z_{k}-h(X_{k/k-1}^{i})-r_{k}$$

(41) $$P_{zz,k|k-1}^{i}=h\left(X_{k/k-1}^{i}\right)h^{T}\left(X_{k/k-1}^{i}\right)-Z_{k}Z_{k}^{T}$$

From Equations (38)–(41), the time-varying non-Gaussian measurement noise $R_{k}$ can be estimated by every tracking step as ${\hat{R}}_{k}$.

4. Weight calculation

According to the theory of PF, the weight of every particle can be represented as:

(42) $$w_{k}^{i}=w_{k-1}^{i}\frac{p\left(z_{k}|x_{k}^{i}\right)p\left(x_{k}^{i}|x_{k-1}^{i}\right)}{q\left(x_{k}^{i}|x_{k-1}^{i},z_{k}\right)}$$

where $p\left(x_{k}^{i}|x_{k-1}^{i}\right)$ is the prior probability, and $q\left(x_{k}^{i}|x_{k-1}^{i},z_{k}\right)$ is the importance density function.

According to ref. [31], $q\left(x_{k}^{i}|x_{k-1}^{i},z_{k}\right)$ is always selected as the same as $p\left(x_{k}^{i}|x_{k-1}^{i}\right)$, which leads to:

(43) $$w_{k}^{i}=w_{k-1}^{i}p\left(z_{k}|x_{k}^{i}\right)$$

From Equation (43), the weight of every particle can be computed by $p\left(z_{k}|x_{k}^{i}\right)$ recursively. According to the online estimated measurement noise by Equation (39), $p\left(z_{k}|x_{k}^{i}\right)$ can be represented as:

(44) $$p\left(z_{k}|x_{k}^{i}\right)={\left(2\pi R_{k}\right)}^{-1/2}\exp\left\{-\frac{1}{2}\left(Z_{k}-h\left(X_{k/k-1}^{i}\right)\right)R_{k}^{-1}\left(Z_{k}-h\left(X_{k/k-1}^{i}\right)\right)\right\}$$

From Equations (43) and (44), the weight of i-th particle can be computed. Then, a normalization procedure is performed by the following equation:

(45) $${\tilde{w}}_{k}^{i}=\frac{w_{k}^{i}}{\sum_{i=1}^{n}w_{k}^{i}}$$

5. Measurement Update

According to Equations (37) and (45), the measurement updated state based on Monte Carlo theory can be computed as:

(46) $${\hat{X}}_{k/k}=\sum_{i=1}^{n}w_{k}^{i}{\hat{X}}_{k/k-1}^{i}$$

6. Resampling

In order to avoid the particle degradation phenomenon and make the whole tracking process robust, the resampling method is adopted to generate new particles based on the weights calculated by Equation (45). The principle of the resampling method is to duplicate the more likely particles and cut off the less likely ones, which will lead to some same particles in the new particle system [31]. Before performing the resampling process, the following index needs to be computed:

(47) $${\hat{N}}_{eff}=\frac{1}{\sum_{i=1}^{n}{\left({\tilde{w}}^{i}\right)}^{2}}$$

If ${\hat{N}}_{eff}$ is lower than the preset threshold, the resampling process needs to be performed to generate new particles for the next tracking step as ${\left\{{\hat{X}}_{k}^{i}\right\}}_{i=1,2\cdots n}$ and the weights of the new set of resampled particles are set to be as equally as $\frac{1}{n}$.

7. Process noise online estimation

The 1-st order momentum of the time-varying non-Gaussian noise at the current tracking time can be updated online by the modified Sage-Husa online noise estimator as:

(48) $${\hat{q}}_{k}=\left(1-d_{k}\right){\hat{q}}_{k-1}+\frac{d_{k}}{n}\sum_{i=1}^{n}\left({\hat{X}}_{k/k}-\Phi_{k/k-1}{\hat{X}}_{k|k-1}^{i}\right)$$

8. Time propagation

The tracking time will be propagated to k+1, and the APF will run into step (2) in the (k+1)-th tracking step by the new particle sets computed in step (6) until the whole tracking period is completed. The pseudo-code is listed as Algorithm 1.

Three-dimensional passive underwater target tracking algorithm for the time-varying non-Gaussian environment by distributed underwater buoys

Considering both the optimal topology design criterion by Section 3 and the designed APF, the 3D passive underwater target tracking algorithm for the time-varying non-Gaussian environment by distributed underwater buoys can be summarized by the following steps as Algorithm 1:
**Algorithm 1:** 3D passive underwater target tracking algorithm for the time-varying non-Gaussian environment by distributed underwater buoys**1. Optimal topology determination** 1 Use every two out of three measurements from the buoys to form the observable measurements sets as (1,2), (1,3) and (2,3); 2 Calculate the accuracy index $T_{acc}$ of every topology of the distributed buoys by Equation (14); 3 Calculate the robust index $T_{ob}$ of every topology of the distributed buoys by Equation (18); 4 Use the results from step 1 and 2 to calculate the overall index $T_{all}$ by Equation (19); 5 With the preset index α, determine the optimal topology of the distributed underwater buoys of tracking time k by Equation (20). **2. Tracking the underwater uncooperative target by APF under the time-varying non-Gaussian environment** 1 Based on the determined sets of measurements at tracking time k, initialize the first step particles and the relative weights; 2 Particles are time-updated by Equation (37); 3 Online noise estimation by Equation (39); 4 New weights are calculated by Equation (45); 5 Resampling procedure are checked and performed by Equation (47) and resampling method introduced by ref. [31]; 6 Estimate the current process noise by Equation (48); 7 Time propagation to run the whole algorithm at tracking time k+1.

## 5. Simulations and Discussions

### 5.1. Simulation Scenario

In this section, comprehensive simulations are made to verify the proposed 3D underwater uncooperative target passive tracking algorithm. In addition, by setting the reference coordination, the coordinates of every buoy of the distributed system and the target are represented in the reference coordination. The detailed configuration of the distributed buoys is set out in Table 1. In this paper, we assume that all the underwater buoys have the same sensing and communication range, and can perform the proposed algorithm.

In the simulations, the underwater target is supposed to perform in the CV motion mode with the actual initial state as $X_{0}=\left[\begin{array}{cccccc} 3000 & 3000 & 100 & 4 & -3 & 0 \end{array}\right]$. In addition, the initial guesses and the relative covariance of the algorithm is set as ${\hat{X}}_{0}=\left[\begin{array}{cccccc} 0 & 0 & 0 & 0 & 0 & 0 \end{array}\right]$ and $P_{0}=\mathrm{diag}\left(\begin{array}{cccccc} 10000 & 10000 & 10000 & 1000 & 1000 & 100 \end{array}\right)$, respectively. The time-varying non-Gaussian process noise is modeled as:

(49) $$W_{k}={\left[\begin{array}{cccccc} 0 & 0 & 0 & w_{vx} & w_{vy} & w_{vz} \end{array}\right]}^{T}$$

with

(50) $$\begin{array}{l} w_{vi}=w_{vi,1}+v_{vi,2}\\ {\dot{w}}_{vi,2}=e_{w}\left(i=x,y,z\right) \end{array}$$

where $w_{vi,1}$ is the zero-mean Gaussian white noise with the variance matrix of $E\left(w_{vi,1}w_{vi,1}^{T}\right)=0.01$. In addition, $e_{w}$ is the zero-mean Gaussian white noise with the variance matrix of $E\left(e_{w}e_{w}^{T}\right)=0.01$.

It is also assumed that all the underwater buoys have the same stochastic process of the time-varying non-Gaussian measurement noise and can be modeled as:

(51) $$V_{i,k}={\left[\begin{array}{cc} v_{\theta } & v_{\varphi } \end{array}\right]}^{T}$$

with:

(52) $$\begin{array}{l} v_{\theta }=v_{\theta ,1}+v_{\theta ,2}\\ {\dot{v}}_{\theta ,2}=e_{\theta } \end{array}$$

and

(53) $$\begin{array}{l} v_{\varphi }=v_{\varphi ,1}+v_{\varphi ,2}\\ {\dot{v}}_{\varphi }=e_{\varphi } \end{array}$$

where $v_{\theta ,1}$ and $v_{\varphi ,1}$ are the zero-mean Gaussian white noises with the variance matrix of $E\left(v_{\zeta ,1}v_{\zeta ,1}^{T}\right)=0.25\left(\zeta =\theta ,\varphi \right)$. Furthermore, $e_{\theta }$ and $e_{\varphi }$ are the zero-mean Gaussian white noise with the variance matrix of $E\left(e_{i}e_{i}^{T}\right)=0.01\left(i=\theta ,\varphi \right)$.

All the simulations in this paper are performed using MATLAB R2019b on the computer with the Microsoft Windows 10 system and the computer is configured with Intel(R) Core (TM) i7-9700k CPU @3.2 GHz. The simulation results are the average of 50 Monte Carlo experiments with the particle number of 2000 for every simulation. According to ref. [13], the total simulation time is set as 60 s with a 1 s measurement interval. In order to evaluate the performance of the proposed algorithm, the root mean square error (RMSE) for locations and velocities of the underwater uncooperative target are adopted, respectively, which can be represented as:

(54) $$\begin{array}{l} RMSE_{l}=\sqrt{\frac{1}{N}\sum_{i=1}^{N}\left(\Delta x_{k}^{2}+\Delta y_{k}^{2}+\Delta z_{k}^{2}\right)}\\ RMSE_{v}=\sqrt{\frac{1}{N}\sum_{i=1}^{N}\left(\Delta v_{xk}^{2}+\Delta v_{yk}^{2}+\Delta v_{zk}^{2}\right)} \end{array}$$

with

(55) $$\begin{array}{l} \Delta x_{k}^{}=x_{k}-{\bar{x}}_{k}\\ \Delta y_{k}^{}=x_{k}-{\bar{y}}_{k}\\ \Delta z_{k}^{}=z_{k}-{\bar{z}}_{k} \end{array}$$

and

(56) $$\begin{array}{l} \Delta v_{xk}^{}=v_{xk}-{\bar{v}}_{xk}\\ \Delta v_{yk}^{}=v_{yk}-{\bar{v}}_{yk}\\ \Delta v_{zk}^{}=v_{zk}-{\bar{v}}_{zk} \end{array}$$

where N is the total number of the Monte Carlo trials, $\left(\begin{array}{ccc} x_{k} & y_{k} & z_{k} \end{array}\right)$ and $\left(\begin{array}{ccc} {\bar{x}}_{k} & {\bar{y}}_{k} & {\bar{z}}_{k} \end{array}\right)$ are the real and estimated locations of the underwater target, respectively, along with $\left(\begin{array}{ccc} v_{xk} & v_{yk} & v_{zk} \end{array}\right)$ and $\left(\begin{array}{ccc} {\bar{v}}_{xk} & {\bar{v}}_{yk} & {\bar{v}}_{zk} \end{array}\right)$ are the real and estimated velocities of the underwater target, respectively.

### 5.2. Optimal Topology Design Algorithm Verification

**Figure 2:**
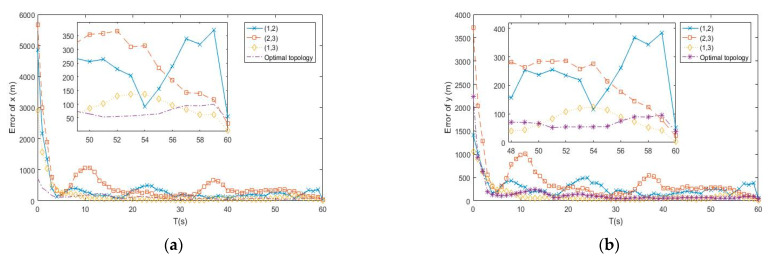
(**a**) Error of x by different topologies of buoys; (**b**) Error of y by different topologies of buoys.

**Figure 3:**
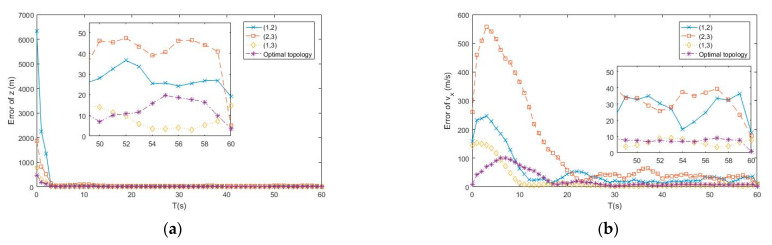
(**a**) Error of z by different topologies of buoys; (**b**) Error of $v_{x}$ by different topologies of buoys.

**Figure 4:**
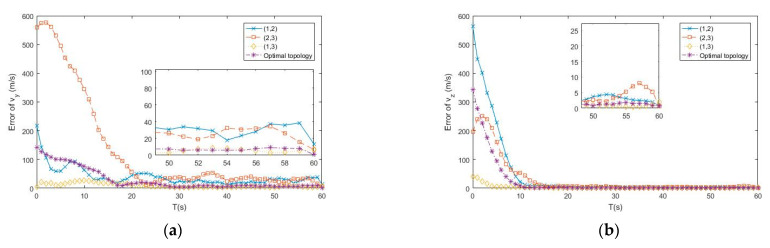
(**a**) Error of $v_{y}$ by different topologies of buoys; (**b**) Error of $v_{z}$ by different topologies of buoys.

In this subsection, the optimal topology design algorithm proposed by Section 3 is tested by the APF tracking algorithm. Figure 2, Figure 3 and Figure 4 show the simulation tracking results of each state by different topology of the distributed underwater buoys, namely (1,2), (1,3), and (2,3), and the online optimal topology is determined by Equation (20), respectively. The $RMSE_{l}$ and $RMSE_{v}$ for the estimating results of different topologies are listed in Table 2. From the simulation results, all the topologies can make converged estimations of the underwater uncooperative target, but the accuracies are different under the specific topology. From Table 2, the $RMSE_{l}$ and $RMSE_{v}$ of the proposed online optimal topology adjustment method have the lowest values. The accuracies of the $RMSE_{l}$ and $RMSE_{v}$ of the online topology adjustment method are 15.48 m and 1.52 m/s, respectively, which is 80.4% and 91.7% higher than the worst estimating results by the fixed topology of buoys as (1,2). It should be noted that the estimation errors of the velocities are much higher than their actual values. This phenomenon occurs because the target is performing in a such low speed which is lower than the lowest location-estimating accuracy. Under this circumstance, the estimations of the velocities can only provide a convergence and more meaningful estimations of the location of the target. Nevertheless, for a slow-moving underwater uncooperative target, the locations are of a higher interest to the observer. Therefore, the idea that better estimations of the velocities lead to superior estimations of the locations of the target still has vital importance. The results prove that the estimations made by the proposed online optimal topology design method has the highest accuracy than the fixed ones, which shows the effectiveness of the proposed optimal topology design algorithm.

### 5.3. APF Verification

**Figure 5:**
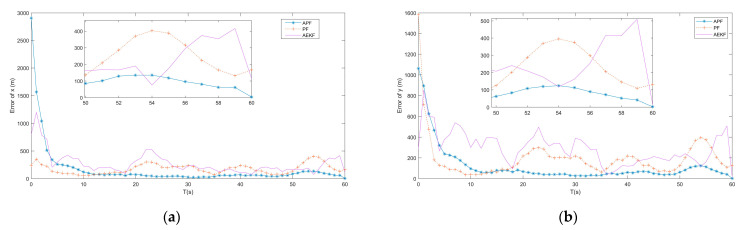
(**a**) Error of x by APF, AEKF and PF; (**b**) Error of y by APF, AEKF and PF.

**Figure 6:**
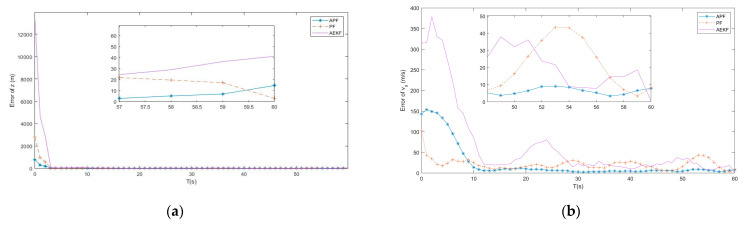
(**a**) Error of z by APF, AEKF and PF; (**b**) Error of $v_{x}$ by APF, AEKF and PF.

**Figure 7:**
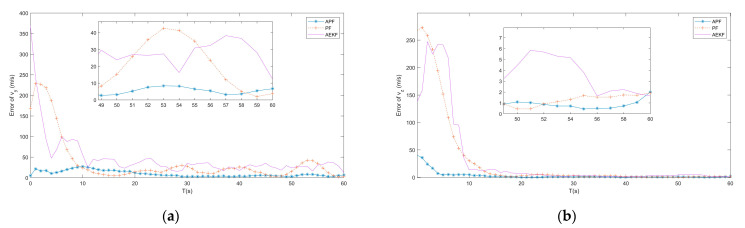
(**a**) Error of $v_{y}$ by APF, AEKF and PF; (**b**) Error of $v_{z}$ by APF, AEKF and PF.

In the subsection, the APF algorithm proposed by Section 4 is tested and compared to the adaptive extended Kalman filter (AEKF) that combined the modified Sage-Husa online noise estimating technique and the EKF, as described in ref. [19], and the traditional PF tracking algorithm, as described in ref. [24], under the same simulation environment. Figure 5, Figure 6 and Figure 7 show the simulation tracking results of every state by the proposed APF, AEKF, and the traditional PF by the same optimal topology design method. In addition, the $RMSE_{l}$ and $RMSE_{v}$ for the proposed APF, AEKF, and the traditional PF are listed in Table 3. Since the APF can calculate the non-Gaussian time-varying noise online, the results are more accurate than the ones made by the traditional PF. In addition, the linearization errors give the AEKF the worst estimating accuracy. From the simulation results, the modified Sage-Husa adaptive technique can make reliable online noise estimation, which further makes the final tracking accuracy higher. In addition, the PF technique can overcome the drawbacks caused by the system nonlinearities.

From the simulation results, the estimating accuracies are enhanced by 92.4% and 83.4% by the proposed APF for $RMSE_{l}$ and $RMSE_{v}$, respectively, when compared to the traditional PF. In addition, it can be found from the simulation results that the convergence time of the APF is much faster than the one of the traditional PF. As a result, the superior performance of the proposed APF is verified by the simulation results.

**Figure 8:**
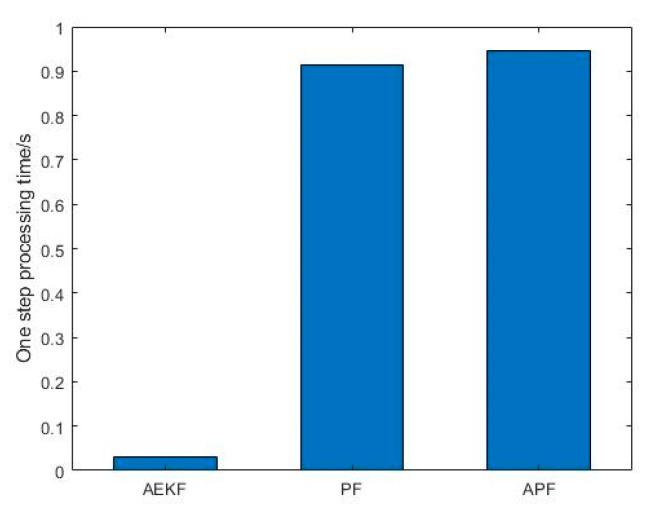
One-step processing time for APF, PF, and AEKF.

The computational complexity of the APF, AEKF, and traditional PF are shown in Figure 8 by representing their one-step processing time. It can be found that the APF and PF have similar one-step processing times since the PF process has the most computational complexity. Although AEKF is easy to operate and has the least computational load, its off-line Jacobian matrix calculation efforts and low tracking accuracy under high nonlinear environments make it the least favorable one to use in our situation.

## 6. Conclusions

The 3D passive tracking of the underwater uncooperative target using distributed underwater buoys for a time-varying non-Gaussian environment is studied in this paper. Firstly, for the sake of accurate tracking and minimum underwater acoustic resource utilization, the optimal topology of the distributed underwater buoys system is designed based on both accuracy theory and observability analysis. Then, based on the determined optimal topology of the distributed underwater buoys at current tracking time, an APF is proposed to tackle the nonlinear underwater target tracking problem with time-varying non-Gaussian noise. From the simulation results, the following conclusions can be drawn. Firstly, the optimal topology design method introduced by this paper can select proper sets of buoys dynamically and can provide the best estimations among all the possible configurations of the distributed buoys. Secondly, by adopting the online noise estimation technique, the APF has significantly higher accuracies compared to the AEKF and the traditional PF with very little extra computational load. It is believed that the APF proposed by this paper has great potential in real-time 3D underwater target passive tracking missions.

## Figures and Tables

**Table 1:** Detailed configuration of the distributed buoys.

Buoy Number	Coordinate
1	$\left(x_{1},y_{1},z_{1}\right)=\left(0,600,50\right)$
2	$\left(x_{2},y_{2},z_{2}\right)=\left(600,0,50\right)$
3	$\left(x_{3},y_{3},z_{3}\right)=\left(0,0,20\right)$

**Table 2:** The $RMSE_{l}$ and $RMSE_{v}$ for different topologies.

	$RMSE_{l}\;(m)$	$RMSE_{v}(m/s)$
(1,2)	79.1	18.41
(1,3)	52.28	10.53
(2,3)	36.97	12.48
Optimal topology	15.48	1.52

**Table 3:** The $RMSE_{l}$ and $RMSE_{v}$ for APF, AEKF and traditional PF.

	$RMSE_{l}\;(m)$	$RMSE_{v}\;(m/s)$
APF	15.48	1.52
PF	211.5	9.18
AEKF	230.5	13.31

## Data Availability

Data is contained within the article.
